# Supplementary material for: Long-Term Follow-Up of Inpatients with Failed Back Surgery Syndrome Who Received Integrative Korean Medicine Treatment: A Retrospective Analysis and Questionnaire Survey Study
Source: J Clin Med. 2021 Apr 15;10(8):1703. doi: 10.3390/jcm10081703 (PMC8071221; doi:10.3390/jcm10081703)
Supplement: Supplementary file 1 [file jcm-10-01703-s001.zip › jcm-1125157-supplementary.pdf]

Journal of
Clinical Medicine

Table S1. Characteristics of patients with FBSS.

Characteristics	Respondent (*n* = 106)	Non-respondent (*n* = 128)	*p*-value
**Age**			
Mean ± SD	53.8 ± 11.9	55.8 ± 11.1	0.171
20≤, <30	2 (1.9)	1 (0.8)	0.562
30≤, <40	15 (14.2)	12 (9.4)	
40≤, <50	23 (21.7)	26 (20.3)	
50≤, <60	23 (21.7)	31 (24.2)	
60≤, <70	40 (37.7)	49 (38.3)	
70≤	3 (2.8)	9 (7.0)	
**Sex**			
Male	35 (33.0)	58 (45.3)	0.075
Female	71 (67.0)	70 (54.7)	
**Length of hospital stay**			
Mean ± SD (day)	30.5 ± 17.9	26.1 ± 16.1	0.051
Median (IQR)	26.5 [17 – 39.8]	21.5 [13 – 38]	
**Type of spinal surgery**[1]			
Laminectomy	95 (89.6)	114 (89.1)	1
Discectomy	15 (14.2)	16 (12.5)	0.859
Spinal fusion	32 (30.2)	39 (30.5)	1
Vertebroplasty	2 (1.9)	0 (0.0)	0.397
Artificial disc replacement	5 (4.7)	7 (5.5)	1
**Operated disc levels**[1]			
L1-2	2 (1.9)	7 (5.5)	0.282
L2-3	5 (4.7)	8 (6.2)	0.824
L3-4	17 (16.0)	19 (14.8)	0.944
L4-5	73 (68.9)	84 (65.6)	0.7
L5-S1	43 (40.6)	41 (32.0)	0.223
**Onset**			
Mean ± SD (day)	246.3 ± 573.8	169.7 ± 394.1	0.229
Median (IQR)	46 [7 – 153]	38.5 [7 – 122]	
**Mode of onset**			0.481
No specific cause	69 (65.1)	81 (63.3)	
Overwork / Over exercise	16 (15.1)	26 (20.3)	
Trauma / Fall	17 (16.0)	16 (12.5)	
Other	4 (3.8)	3 (2.3)	
Unknown	0 (0.0)	2 (1.6)	
**Analgesics**[†]			
Mean ± SD	2.9 ± 10.6	4.2 ± 10.4	0.319
Median (IQR)	0 [0 – 0]	0 [0 – 6.3]	
**Steroid injections**[†]			
Mean ± SD	1.1 ± 5.0	0.8 ± 1.3	0.559
Median (IQR)	0 [0 – 1]	0 [0 – 1]	
**Radiological findings of MRI/CT scans**[1]			
Herniation of the nucleus pulposus	61 (57.5)	68 (53.1)	0.586
Protrusion	40 (37.7)	37 (28.9)	0.197
Extrusion	34 (32.1)	43 (33.6)	0.915
Sequestration	0 (0.0)	0 (0.0)	—
Spinal stenosis	31 (29.2)	36 (28.1)	0.965
Central canal	18 (17.0)	24 (18.8)	0.857
Foraminal	20 (18.9)	22 (17.2)	0.871
Spondylolisthesis	10 (9.4)	7 (5.5)	0.363
Vertebral fracture	5 (4.7)	6 (4.7)	1
Other	1 (0.9)	1 (0.8)	1
**Comorbidity**[1]			
Hypertension	22 (20.8)	15 (11.7)	0.088
Diabetes mellitus	10 (9.4)	12 (9.4)	1

Cardiovascular disease	23 (21.7)	21 (16.4)	0.388
Thyroid-related comorbidity	4 (3.8)	0 (0.0)	0.087
Liver-related comorbidity	3 (2.8)	7 (5.5)	
Other	27 (25.5)	36 (28.1)	
**Smoking**			0.068
Yes	14 (13.2)	30 (23.4)	
No	92 (86.8)	98 (76.6)	
**Alcohol intake**			0.66
Yes	20 (18.9)	24 (18.8)	
No	86 (81.1)	103 (80.5)	
Unknown	0 (0.0)	1 (0.8)	
**Occupation**			0.466
Unemployed‡	62 (58.5)	71 (55.5)	
Office work§	25 (23.6)	38 (29.7)	
Service or retail industry[11]	13 (12.3)	9 (7.0)	
Manual labour††	6 (5.7)	9 (7.0)	
Unknown	0 (0)	1 (0.8)	
**NRS score of LBP at admission**	5.9 ± 1.4	5.7 ± 1.3	0.368
**NRS score of leg pain at admission**	4.6 ± 2.6	4.3 ± 2.9	0.397
**ODI score at admission**	51.1 ± 15.7	50.1 ± 17.3	0.626
**EQ5D score at admission**	0.5 ± 0.2	0.5 ± 0.2	0.634

[1] Multiple check.† Treatments before admission.‡ Housewife/student/retired.§ Office worker/manager/public servant/professional practitioner.[11] Self-employed/service or retail industry worker.†† Agriculture, forestry, fishery, or mining industry worker/equipment mechanic or machinery operator/professional soldier.FBSS: Failed back surgery syndrome; SD: Standard deviation; IQR: Interquartile range; MRI: Magnetic resonance imaging; CT: Computed tomography NRS: Numeric rating scale; LBP: Low back pain; ODI: Oswestry disability index; EQ-5D: EuroQol 5-dimension.

Table S2. Type of integrative Korean medicine treatment (n = 234).

	N (%)	Mean ± SD
Herbal medicine	232 (99.1%)	82.0 ± 50.6
Acupuncture	234 (100%)	52.1 ± 32.1
Pharmacopuncture	234 (100%)	51.2 ± 32.3
Chuna manual therapy	220 (94.0%)	26.1 ± 16.9
Traction therapy	37 (15.80%)	20.5 ± 13.6
Hot packs	149 (63.70%)	19.2 ± 16.4

SD: Standard deviation.
